# Medical secretaries’ fears and opportunities in an increasingly digitalised workplace environment

**DOI:** 10.1108/JHOM-04-2023-0127

**Published:** 2024-04-30

**Authors:** Maria Qvarfordt, Stefan Lagrosen, Lina Nilsson

**Affiliations:** Department of Medicine and Optometry, eHealth Institute, Linnaeus University, Kalmar, Sweden; Department of Management, School of Business and Economics, Linnaeus University, Kalmar, Sweden

**Keywords:** Medical secretary, Healthcare digitalisation, Organisational change, Administrative staff, Digital transformation

## Abstract

**Purpose:**

The purpose of this mixed-methods study was to explore how medical secretaries experience digital transformation in a Swedish healthcare organisation, with a focus on workplace climate and health.

**Design/methodology/approach:**

Data were collected using a sequential exploratory mixed-methods design based on grounded theory, with qualitative data collection (a Quality Café and individual interviews) followed by quantitative data collection (a questionnaire).

**Findings:**

Four categories with seven underlying factors were identified, emphasising the crucial need for effective organisation of digital transformation. This is vital due to the increased knowledge and skills in utilising technology. The evolving roles and responsibilities of medical secretaries in dynamic healthcare settings should be clearly defined and acknowledged, highlighting the importance of professionality. Ensuring proper training for medical secretaries and other occupations in emerging techniques is crucial, emphasising equal value and knowledge across each role. Associations were found between some factors and the health of medical secretaries.

**Research limitations/implications:**

This study adds to the knowledge on digital transformation in healthcare by examining an important occupation. Most data were collected online, which may be a limitation of this study.

**Practical implications:**

Several aspects of the medical secretaries’ experiences were identified. Knowledge of these is valuable for healthcare managers to make digital transformation more effective while avoiding excessive strain on medical secretaries.

**Originality/value:**

Medical secretaries are expected to contribute to the digitalisation of healthcare. However, minimal research has been conducted on the role of medical secretaries in workplace digitalisation, focusing on workplace roles and its dynamics.

## Introduction

Digitalisation profoundly affects workplace settings across a range of sectors (Harteis, 2018; Sætra and Fosch-Villaronga, 2021). Extant research has reported how digitalisation influences workplace routines, employee learning processes and skill development and requirements (Gjellebæk *et al.*, 2020). Historically, digital services and technologies were embraced in Sweden before widespread adoption in the public healthcare organisations which were perceived as slower in deploying digitalisation than sectors such as banking (Øvretveit, 2019). Currently, various digital services and technologies are implemented and used in the Swedish healthcare system. Many digitalisation efforts are limited to smaller pilot schemes or provided independently within private initiatives. From an organisational perspective, healthcare digitalisation enhances collaboration (Bossen *et al.*, 2014), streamlining care pathways. However, it may also challenge established (power) structures, potentially threatening traditional roles (Bossen *et al.*, 2014).

Digitalisation shows promise in addressing the challenge posed by an increasing number of patients amid shortages of healthcare professionals (Blease *et al.*, 2018; Lai *et al.*, 2020). However, the complexity of transforming the Swedish healthcare system through digitalisation becomes evident when navigating between newly introduced and traditional methods, presenting challenges and potentially generating dynamic tension in workplace settings (Östlund, 2017). From the perspectives of medical secretaries, tasks introduced amid this complex organisational change (Star and Strauss, 1999) may be understood through the lens of “articulation work”, encompassing efforts to navigate and address unforeseen challenges not only in individual technology use but also within collaborative or group settings (Strauss, 1985).

Furthermore, some healthcare professionals’ current administrative tasks may be managed (or at least assisted) by technology (De Maeseneer *et al.*, 2019) or removed from the workflow routine (Erickson *et al.*, 2017). However, the digitalisation in healthcare should be adopted thoughtfully (Butcher and Hussain, 2022) and might be implemented more successfully with active employee involvement (Garmann-Johnsen *et al.*, 2020; Gjellebæk *et al.*, 2020). Therefore, to comprehend the impacts of digitalisation on healthcare workplaces, it is essential to first understand the impact of digitalisation on the tasks performed and the workforce. However, the effects are often unpredictable (Barley, 2020).

There is growing evidence on how clinicians experience healthcare digitalisation (Laukka *et al.*, 2020; Shinners *et al.*, 2020). Nonetheless, in light of rapid technological progress, more in-depth evidence is required to explore effects of healthcare digitalisation on workplace settings (Sætra and Fosch-Villaronga, 2021), particularly regarding non-clinical occupations (Bossen *et al.*, 2012) such as secretaries (Holten Møller and Vikkelsø, 2012). Medical secretaries play an essential role in ensuring regularity, workflow and serviceability in today’s healthcare system. As a non-clinical healthcare occupation, they are essential when implementing healthcare digitalisation processes (Bossen *et al.*, 2012). However, there is a lack of evidence regarding how the role of medical secretary is characterised as an occupation. Medical secretaries, among other non-clinical healthcare staff, seem to be underrepresented in research (Bossen *et al.*, 2012; Karlsson, 2009). Further research is thus needed on medical secretaries’ experiences of digital transformation and the occupation’s level of influence over these changes (Zuin and Findlay, 2014). In accordance with previous recommendations to broaden the understanding of what medical secretaries do (Bossen *et al.*, 2012) and what kind of workplace changes they face (Zuin and Findlay, 2014), the purpose of this mixed-methods study was to explore how medical secretaries experience digital transformation in a Swedish healthcare organisation, with a focus on workplace climate and health.

## Theoretical concepts

### Medical secretaries

The exact origin of the medical secretary occupation is difficult to determine in terms of time and place. However, at the end of the 19th century, it was documented that administrative tasks were carried out by “administrative staff” in European healthcare settings (Tyler and Cummins, 2004). The medical secretary occupation was officially known as the doctors’ secretary who provided administrative ease for doctors (Bertelsen and Nøhr, 2006). In today’s healthcare system, the medical secretary plays a crucial role (Medford, 2013; Hooke, 2016) in supporting the workflow, regularity and serviceability of the healthcare system (Medford, 2013). By performing administrative tasks medical secretaries enable clinicians to devote more time to their patients (Kennedy, 2016), thereby contributing to the efficiency of workplace routines (Hooke, 2016).

The core tasks of medical secretaries include handling a variety of paperwork, such as filing and editing documents (Alis and Blair, 2003; Mohr *et al.*, 2003; Laerum *et al.*, 2004), printing clinical information (Reddy and Spence, 2008), transcribing and filing clinicians’ dictations (Bossen and Markussen, 2010), locating files (Reddy and Jansen, 2008), staffing the reception (Schmidt *et al.*, 2007) and providing general clerical support, often in a collaborative manner between occupations (Bossen and Markussen, 2010; Reddy and Jansen, 2008). The work routines of medical secretaries may also include patient contact and handling of clinical test results of patients (Alis and Blair, 2003). Medical secretaries play a crucial role as a resource in diagnostic work, placing their responsibilities at the forefront (Holten Møller and Vikkelsø, 2012). Ensuring the quality (Johansen *et al.*, 2015) and completeness (Bossen *et al.*, 2012) of documentation is also part of their tasks (Johansen *et al.*, 2015), and applying the clinic-specific knowledge when in the contact with patients (Agrawal *et al.*, 2020), as well as when transcribing clinician’s dictations (Bertelsen and Nøhr, 2006).

Previous research indicates that medical secretaries are the bridge between the systematic delivery of healthcare and healthcare delivery as a “service”, suggesting that the occupation is needed for much more than just transcribing dictations and “paperwork” (Hooke, 2016; Morgan, 2022). Moreover, the digitalisation of healthcare is anticipated to heighten the significance of the medical secretary occupation (Bossen *et al.*, 2014), prompting a need for a re-evaluation of the occupation’s role (Morgan, 2022). Within this context of digital transformation, medical secretaries play a pivotal role in enhancing patient care, particularly through their active participation in multidisciplinary teams (Agrawal *et al.*, 2020). However, optimising digitalisation strategically with time and training allocation is crucial when further elevating their role parallel to its ongoing progress (Morgan, 2022).

### Shift in healthcare tasks

Some forms of digitalisation in healthcare entail transforming paper-format documents into digital versions (digitisation) (Bhavnani *et al.*, 2016), whereas more complex changes involve digital devices performing tasks in support of humans (digitalisation) (Bhavnani *et al.*, 2016). Several digitalisation initiatives require the development of new skills to keep pace with and conform to new technologies (Bossen *et al.*, 2014).

These changes have directly impacted medical secretaries’ work routines (Bertelsen and Nøhr, 2006), because they include being introduced to new responsibilities, as a consequence of the removal or decrease of some tasks, and coping with new tasks. Although medical secretaries’ work has been affected by digitalisation over the last two decades (Bossen *et al.*, 2014), current changes include adapting to an increasingly dynamic work environment caused by the shift of tasks from some occupations to others (Bossen *et al.*, 2014). Specific aspects that have caused work tasks to be added or changed for medical secretaries may include the use of electronic information systems (Laerum *et al.*, 2004; Santavirta *et al.*, 2021), electronic medical records (Laerum *et al.*, 2004) and the implementation of voice recognition technology for digital transcriptions of healthcare professionals’ dictations (Hodgson and Coiera, 2016; Parente *et al.*, 2004).

### Workplace health promotion

A salutogenic perspective on promoting health in the workplace underscores the idea that workplaces have the potential to provide advantageous settings for improving the health and well-being of employees during work (Antonovsky, 1987) by using the imbedded concept “Sense of Coherence” (SOC) (Antonovsky, 1987, 2002). Comprehensibility, manageability and meaningfulness are the three components of SOC that influence health. Individuals construct their SOC through life experiences, which are general resistance resources such as resources in work life. Quality of life, health and job satisfaction are positively correlated with having strong SOC, and this concept has previously been used to explore well-being in the healthcare sector (Nilsson *et al.*, 2012).

The SOC theory has previously been proven useful in addition to workplace health promotion, since successful application of SOC components was used in research exploring healthcare workers’ health (Nilsson *et al.*, 2012). Enhanced health and well-being can be achieved by promoting strong SOC in workplace settings (Bakker and Demerouti, 2007), as the concept is closely tied to employees’ perspectives on their work climates (González-Siles *et al.*, 2022). Regarding the healthcare sector, the digitalisation of workplaces may result in emotional disengagement among employees, posing challenges to the meaningfulness of work and potentially hindering social exchanges (Palumbo, 2022). Additionally, work-life health is influenced by a satisfactory work climate, the ability to influence the work situation and sufficient equipment (Kira and Forslin, 2008).

## Methodology

This study was conducted in the public healthcare organisation of a county in southern Sweden. The organisation includes hospitals, primary healthcare centres and special care centres. Consistent with the grounded theory employed in this study, the selection of methods evolved incrementally as data collection advanced, resulting in a study design that did not adhere strictly to a linear pattern (Chun Tie *et al.*, 2019). Instead, it developed iteratively as the research progressed, without explicit alignment with any specific theoretical orientation. This iterative process is in accordance with the “mixed grounded theory” (MGT) (Creamer, 2021; Johnson and Walsh, 2019), which incorporates grounded theory (GT) (Glaser and Strauss, 2006) and mixed methods (Creswell and Plano Clark, 2017). Hence, to use mixed methods and GT simultaneously, owing to our evolving methodological standpoints (Creamer, 2021; Johnson and Walsh, 2019), we utilised the MGT methodological framework.

Data collection followed an exploratory sequential mixed-methods design (Creswell and Plano Clark, 2017). First, a Quality Café (Lagrosen, 2017) was conducted to collect the qualitative first-phase data. The results from the Quality Café were used to develop an interview guide for collecting the qualitative second-phase data using semi-structured individual interviews (Patton, 2014) with open-ended questions (Britten, 1995). The sequence of this process was driven by the belief that research group discussions capitalise on the dynamic nature of groups that may foster dialogues that explore the interplay between interpersonal relationships. In contrast, individual interviews can potentially yield more in-depth information from specific respondents (Kidd and Parshall, 2000). Given these recognised differences, this chosen design aimed to capitalise on the complementary strengths inherent in each method, with the method producing more “surface” data (Powell and Single, 1996) being initially utilised.

Findings from both qualitative phases were initially analysed separately using thematic analysis, which offers a systematic approach to the analysis of qualitative interview data when used in conjunction with GT (Chapman *et al.*, 2015). Furthermore, as previously suggested, the results from the two independently analysed datasets were compared to detect similarities and construct an integrated synthesis (Cronin *et al.*, 2008). Our main priority was to emphasise the experiences and concerns of participants, thus developing a theorem from the data without pre-existing conceptions, which is in line with GT (Glaser and Strauss, 2006).

The questionnaire for quantitative data collection was based on the qualitative findings. The quantitative phase of the present study had a twofold purpose. First, we wanted to confirm the qualitative findings by including organisational and workplace-related items in line with the interview guide. Second, the topic of health was introduced as part of the questionnaire in accordance with the collection of additional evidence in the iterative process of MGT (Creamer, 2021; Johnson and Walsh, 2019). This study was approved by the Swedish Ethical Review Authority (2021–01318) and followed COREQ reporting guidelines (Tong *et al.*, 2007).

### Participant eligibility and recruitment

The inclusion criterion for all phases of the study was employment as a medical secretary within the public healthcare organisation of the county. Purposeful sampling was used as this approach is suitable when insights from a specific group are desired (Campbell *et al.*, 2020). Regarding the individual interviews, information was sent to all medical secretaries in the organisation (approximately 300) via email in late February 2022.

Medical secretaries who showed interest in participating received further written information about the study, its purpose, and what participation in the study entailed with respect to ethical considerations and consent, as well as suggestions for interview appointments via email from one of the researchers. Twenty-four medical secretaries agreed to participate in the study, however, owing to time limitations, it was not possible to arrange interviews for four of them; therefore, 20 interviews were finally conducted.

## Data collection

### Quality Café

In October 2021, a Quality Café (Lagrosen, 2017) was held with 14 medical secretaries from two clinics. The researchers of the present study were hosts of the Quality Café, which was held in Swedish for three hours. The Quality Café was originally designed to integrate the World Café technique with quality improvement methods for the purpose of facilitating conversations on a main topic (Lagrosen, 2017). The topic of the present study was “In what way could medical secretaries contribute to the development of eHealth?”.

After an introduction, the participants were divided into groups of four or five. This was because a Quality Café comprises group sessions (three in total) wherein participants discuss the topic. Each group session lasted approximately 30 min, and the participants regrouped prior to every new session. One participant at each table was the host for the group sessions. Therefore, this person remained at a specific table during all sessions and took notes on the discussion. The notes were later presented by the hosts.

Next, all participants were divided into two equally sized groups. Each group had access to a flipboard that allowed the participants to create and organise a collaborative affinity diagram that emerged iteratively during the process. After creating the two affinity diagrams, all participants gathered for a final session, wherein the content was presented with a following concluding discussion. Two researchers documented the entire Quality Café step-by-step to enable content analysis.

### Qualitative individual interviews

Between April and May 2022, 20 interviews were conducted with medical secretaries from all parts of the organisation. One of the interviewees had participated in the quality café, the other 19 had not. Besides background questions, the interview guide comprised three themes that were developed based on the results from the quality café:

1. *Introductory questions on digitalisation* based on general questions regarding workplace digitalisation.
2. *Digitalisation and the medical secretary as an occupation* arising from considerations about the professional roles of medical secretaries, recognising their potential along with a desire for acknowledgement, as well as a shared commitment to organisational progress.
3. *Preconditions for digitalisation,* stemming from the experienced preconditional aspects of well-developed technical solutions, having adequate personal skills along with proper IT support.

The length of the interviews ranged from 42 to 60 min (mean: 51 min). All interviews followed the same interview guide and were conducted via video conference (19 out of 20) or phone calls (1 out of 20). The interviews were recorded using an external audio recorder. The participants received verbal information about the study purpose prior to the recording in accordance with the initially obtained emailed information. The participants provided verbal consent to participate in the study at the beginning of the recording.

The interview guide was pilot-tested with one medical secretary to examine and further develop the interview questions, ensuring its relevance in accordance with Majid *et al.* (2017).

### Questionnaire

The questionnaire comprised 40 items (excluding background questions). The questions were developed based on the results of the qualitative phases. In addition, two three-item indices measuring health that have been used in previous studies were added; the first measured respondents’ self-reported health, and the second contained a short assessment of SOC dimensions.

A Likert scale was used to evaluate participants’ answers. To make the Likert scale more like a continuous scale (which allows arithmetic operations), recommendations to use 11 Likert scale points (Wu and Leung, 2017) ranging from 0 to 11 were followed (Hodge and Gillespie, 2007; Leung, 2011).

## Data analysis

### Quality Café and individual interviews

A thematic analysis, employing an inductive approach, was conducted to analyse the notes from all sessions, affinity diagrams of the Quality Café and transcripts from individual interviews. This analysis process is outlined in six steps, from the initial familiarisation with the data via coding, through the iterative construction of categories and sub-categories, to the final implementation of the analysis in the article (Braun and Clarke, 2006). To arrange the qualitative results more clearly, a coding scheme was constructed based on the data from both qualitative phases.

### Questionnaire

#### Factor analysis

Preparatory data analyses were performed before the main analyses. These included testing whether the data were normally distributed using the Shapiro–Wilk test (Ghasemi and Zahediasl, 2012). As the quantitative data were not normally distributed, a principal axis factoring analysis (Brown, 2015) was performed. Principal axis factoring analysis considers all possible variances, such as errors and common as well as unique variances between items, hence accepting that errors may exist in the data (Tavakol and Wetzel, 2020).

However, in factor analysis, two different rotations are generally suggested to be performed when aiming to examine the dimensionality that underlies the items (variables) chosen. These two rotations, Promax and Varimax, have been suggested to yield similar results for items loading within factors (Finch, 2006). The two rotations were then performed. Compared with the Varimax rotation, the results of the Promax rotation showed more consistency in factor loadings. Minor differences were detected between the two rotation techniques, but were not further considered owing to their slightness.

The Promax rotation accepts factor-loaded items in the analysis as correlated, as seen in the dataset of the present study. Inter-correlation is common in nearly all scientific contexts concerning the study of societies and relationships among individuals (Hair, 2011; Matsunaga, 2010). Hence, only the analysis with the Promax rotation was included. Furthermore, the Promax rotation was used to explain the relationships between items, rather than simply reducing them (Tavakol and Wetzel, 2020).

There is no golden standard for choosing a factor-loading cutoff point. However, 0.32 is commonly applied as a thumb rule, with the items explaining at least 10 % of the variance in their respective factors. Each factor is recommended to have at least three items loading ≥0.32 (Fabrigar *et al.*, 1999; Costello and Osborne, 2005).

#### Non-parametric correlation analysis

As the factors of the 7-structure model were not normally distributed according to the preferred Shapiro–Wilks test (Ghasemi and Zahediasl, 2012), a non-parametric correlation analysis was used to determine correlations between health indices 1 and 2 as dependent variables and factors of the 7-structure model.

## Results

### The qualitative data collection: interviews

Analysis of the qualitative data revealed two main themes divided into four categories (Table 1). As recommended by Braun and Clarke (2006), the identified categories were strongly linked to the data without using a predefined coding framework.

#### Paving the way for digitalisation

All respondents stated that new work tasks and procedures were a growing part of the workday, particularly since the implementation of voice recognition technology. Receiving information ahead of implementing new initiatives was highlighted as crucial, as was feeling like a part of the workplace’s plans and long-term ambitions. All participants agreed that it was essential to have access to information, opportunities to learn new tools and time to adapt. However, most medical secretaries felt that they were the last to receive information about change initiatives in the workplace, often when the implementation of the initiative was already underway.

The importance of having well-anchored motives and plans regarding change initiatives was emphasised among the medical secretaries, given the varying needs between workplaces. Some respondents expressed the sentiment of “take the bad with the good, and just deal with it” as a medical secretary in a public sector workplace affected by digital transformation. The need to involve all occupations and engage all employees in change initiatives regarding digitalisation was emphasised.

#### Acknowledgement and self-empowerment

The respondents reported that preconceptions prevailed regarding the characteristics of medical secretaries and that they were generally not acknowledged as an occupation. A general lack of understanding exists regarding a medical secretary’s function in healthcare and their role in the operative chain of events within a workplace. Nearly all the respondents expressed the need for their role to be acknowledged, both generally, from a societal perspective, and within individual workplaces. Medical secretaries reported that their occupation had been forgotten because their opinions were not considered, for example, regarding workplace changes.

Some respondents experienced a lack of visibility and value compared with other healthcare employees. Workplace culture seemed to prevail, where medical secretaries were not acknowledged to the same extent as clinicians, nor did they gain the same level of respect. Although the organisation’s culture seemed to encourage transdisciplinary collaboration, workplace hierarchy and different professional statuses were reported as possible barriers to including medical secretaries in digitalisation change management.

#### Thoughts and fears

The impact of change initiatives on medical secretaries as an occupation has occasionally provoked despondency and worries about the profession’s future role because of the changing nature of their workdays. Transcription and handling dictations were expressed by almost all respondents as being one of the core tasks of the occupation, which was also associated with professional pride. A great feeling of loss from replacing writing with other tasks was expressed almost unanimously. Instead of transcribing clinicians’ dictations, new tasks, such as proofreading and editing automatically generated documents, have been added to workday routines. Newly introduced tasks did not always lend well to the perception of how the workday looked. Some respondents expressed that the implementation of digitalisation might require increased patient contact owing to the parallel increase in handling phone calls and staffing receptions. However, some respondents perceived the increased patient contact as positive because this added another dimension to the workday.

#### Digitalisation as an enabler

For some participants, digitalisation was an exciting, positive experience, even when it caused an imbalance in the occupation’s intended role. A positive impact on patient safety was highlighted as paper records or other physical documents risked disappearing from the clinic. The reduced number of dictations to transcribe was experienced as stress reduction by some participants.

Some respondents perceived that the introduction of digitalisation could foster an increased crossing of boundaries between occupations, bringing them closer, promoting interprofessional cooperation and making the role of medical secretaries more acknowledged as a consequence of improved professionalisation and, occasionally, individual development. Additionally, a few respondents stated that digitalisation might foster career possibilities because of the broadening of their work.

### Quantitative data collection: questionnaire

The questionnaire received 181 responses (96% women), with a response rate of 64% which was slightly higher than usual online questionnaire average response rates (Wu *et al.*, 2022).

#### Descriptive statistics

The age and workplace of the respondents is summarised in Table 2.

#### Factor analysis

Initially, the Kaiser–Meyer–Olkin (KMO) sample adequacy measure was 0.854, indicating that the sample size was sufficient at excellent levels (Field, 2009). The spherical value of the Bartlett’s test was significant (*p* = 0.000). Bartlett’s test of sphericity should be significant and less than *p* = 0.050 to indicate that the correlation matrix is significantly different from an identity matrix, having the variables not correlated. As the KMO value was close to 1.0, and Bartlett’s test significance value was 0.000, the data were adequate and appropriate to proceed with the reduction procedures (Field, 2009).

Subsequently, factor analysis (principal axis factoring) was conducted. The factor analysis initially included 34 items. However, one of the factors contained only a two-item loading, which was removed according to previous suggestions (Costello and Osborne, 2005). Factor analysis determined that the 33 items of the questionnaire used in the present study comprised a structure with seven factors. Table 3 presents the distribution of the items, factor loadings and dimensions.

#### Non-parametric analyses

Spearman’s rank correlation coefficient analysis was conducted to determine possible associations between the independent variables (the factors identified in the factor analysis) and dependent variables (the health indices). The results revealed significant positive correlations between perceived health and Factors 1, 2 and 5. They also revealed positive correlations between the SOC index and Factors 1, 2, 3, 5 and 7. All items were significantly correlated at moderate levels (Akoglu, 2018, Table 4).

Additionally, a Kruskal–Wallis analysis (Ostertagová *et al.*, 2014) was conducted to identify potential differences among items and health indexes, treating them as independent variables alongside the variables of age. The analysis identified statistically significant differences (*p* < 0.050) between the age groups “35 years or younger” and “51 years or older” for the item “I am rarely tired” within health index 1 and “I often reflect on how my workday will turn out in the future in relation to digitalisation” within Factor 4 (worries and concerns).

## Discussion

**Figure 1:**
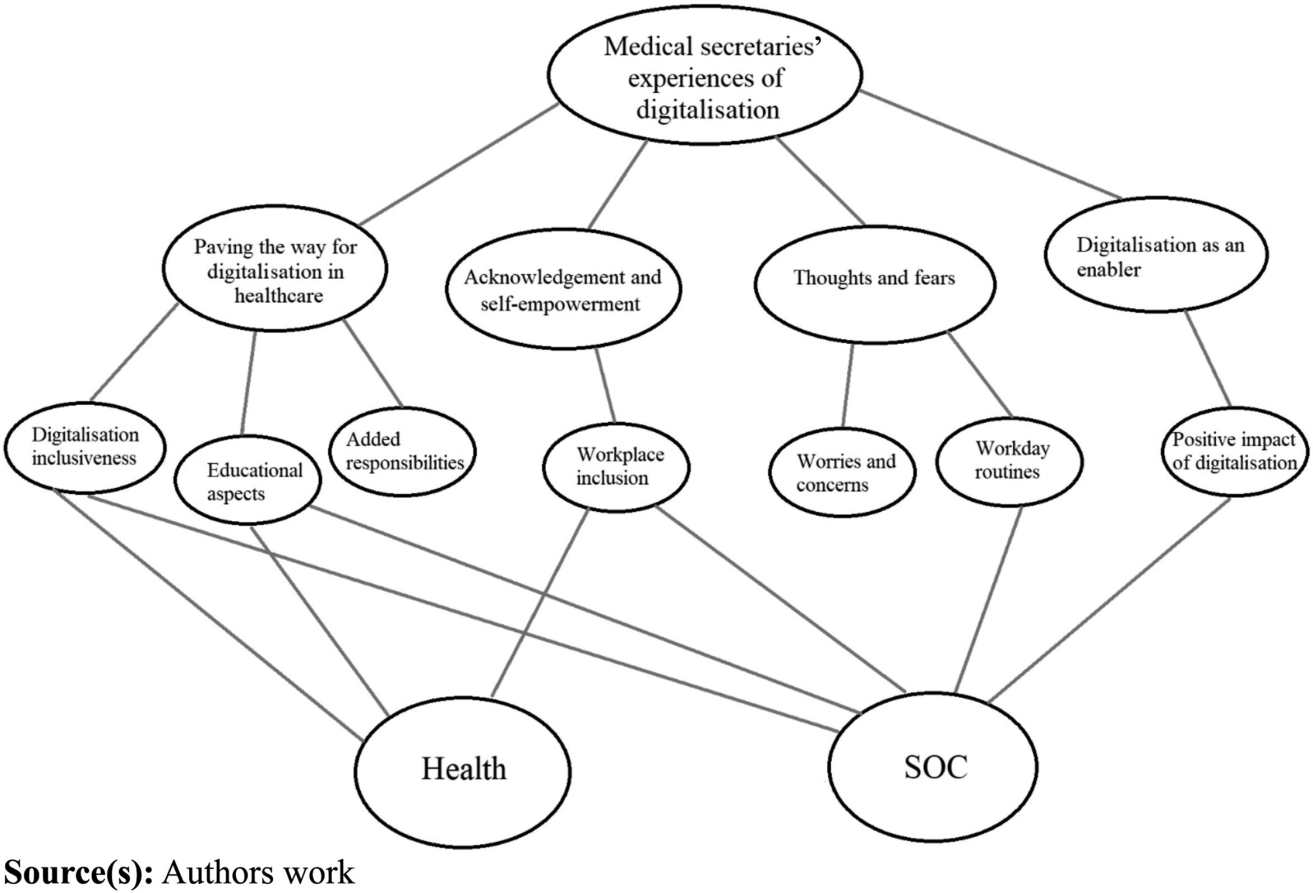
The interaction between health and SOC and the four qualitatively emerged categories along with the 7-factor model as part of the medical secretaries’ experiences of workplace digitalisation

The four qualitatively emerging categories are related to the 7-factor model in Figure 1. The category *Paving the way for digitalisation* is related to the factors *Digital inclusiveness*, *Educational aspects* and *Added responsibility due to digitalisation*. The *Acknowledgement and self-empowerment* category correspond well with *Workplace inclusion*. *Thoughts and fears* can be related to *Worries and concerns* and *Workday routines*. Finally, *Digitalisation as an enabler* corresponds to the *Positive impact of digitalisation*.

Moreover, *Digital inclusiveness, Educational aspects* and *Workplace inclusion* were correlated with the *Perceived health* index. In addition, these three factors, as well as *Workday routines* and *Positive impact of digitalisation,* were associated with the *SOC* index.

Correlation does not imply causation, and even if causation exists, the variable that causes it is uncertain. It is reasonable to assume that a higher sense of coherence should imply a higher ability to handle work changes related to digitalisation. Regarding the correlation with perceived health, the stress of digitalisation may be detrimental to health. Nonetheless, these are merely loose assumptions. This study has shown that there are associations between several aspects of digitalisation and health. Future studies should investigate the causality and mechanisms of these associations.

The adaptions to new work tasks seen in this study correspond to previous findings that medical secretaries are expected to be technologically literate (Côté *et al.*, 2005) and skilled in administration (Lambe *et al.*, 2018). However, new healthcare technologies require new skills and training (Pope and Turnbull, 2017). This may be important because job satisfaction and task performance influence workplace well-being and happiness (Fisher, 2010).

The findings revealed that perceiving digitalisation as something positive was correlated with health. Previous research suggests that employee well-being mediates the relationship between health and digitalisation in workplace settings (Sun *et al.*, 2022). Additionally, collaboration among employees at workplaces was previously found to be associated with improved health (Suter *et al.*, 2012). For positive digitalisation, relevant information may help accept new tasks (Gardner *et al.*, 2010).

Feelings of inclusion were also correlated with health, which may correspond to an inclusive workplace climate that promotes organisational commitment, job satisfaction, individual empowerment and positive health outcomes, as identified earlier (Groggins and Ryan, 2013; Hofhuis *et al.*, 2012). In addition to assuming tasks that may be perceived as inconspicuous (Bergey *et al.*, 2019), medical secretaries have been suggested to feel invisible, particularly in relation to organisational change, which may stem from their work being typically seen as routine and not knowledge-based (Barley, 1996). Previous research suggests that involvement and influence, along with engagement, psychological support, organisational culture and clear expectations of staff, are correlated with employee health (Tsuno *et al.*, 2018). Thus, involving employees in the process of implementing workplace technology, including the implementation rationale, is critical (Williams and Dickinson, 2010). Previous findings have pointed to low participation and influence on the change processes of medical secretaries. This may affect employee health (Lamontagne *et al.*, 2014).

Earlier research also suggests that organisational advancements enable, if not require, a collaborative process across all occupations in healthcare (Bossen *et al.*, 2014). Corresponding to our findings, the growing use of technology in healthcare workplaces may affect secretarial tasks, and these aspects of work life may not be consistently aligned with established work practices and workflows (Bergey *et al.*, 2019). In addition, a closer collaboration between occupations may be prompted by technology use (Bergey *et al.*, 2019; Bossen *et al.*, 2014). Previous research has also indicated that job satisfaction is higher in closed than in open hospital units (Khokher *et al.*, 2009). However, inter-employee collaboration may bridge digital exclusion in workplaces, which, in turn, may impact workplace health (Wissemann *et al.*, 2022).

It has previously been suggested that administrative tasks can be redistributed from healthcare professionals, such as doctors and nurses, to medical secretaries without significant effort (De Maeseneer *et al.*, 2019). Additionally, previous findings suggest that medical secretaries may be expected to perform tasks for which no other employee is responsible yet being an occupation of importance for the workflow (Medford, 2013). It is also suggested that medical secretaries perform tasks beyond their “core missions” such as managing patient-related manners in addition to clinical work (Holten Møller and Vikkelsø, 2012). This is in line with the results of the present study; however, while having a vital role in workplace processes, their position is not always perceived as receiving proper attention.

## Conclusion

In this study, four categories depicting medical secretaries’ experiences of digitalisation were defined using qualitative methods. These were further elaborated on in the quantitative analysis, which identified seven underlying factors. Taken together, these findings show that there are fears and worries among medical secretaries regarding the changes that have taken place and potentially will occur in their occupation. However, the participants also pointed out the positive aspects of digitalisation, seeing it as an enabler of more efficient healthcare and more varied and interesting tasks. An association between medical secretaries’ experiences of digitalisation and their health was also found. Consequently, the findings show that organising and paving the way for digital transformation in an appropriate manner is crucial. Medical secretaries should be involved in this process to ensure that their professional capacity is respected and utilised in the best possible way. In addition, a general acknowledgement of the professionalism of medical secretaries is vital to underline their status in the healthcare system.

## Practical implications

The future roles and responsibilities of medical secretaries employed in highly dynamic healthcare workplaces should be of great interest to management, to bring clarity regarding their positions and functions, including all occupations equally, as part of the workplace structure. Furthermore, providing medical secretaries, among other employees, with proper training in evolving techniques and tasks, should be realised. This could be accomplished by providing healthcare staff with an adequate amount of information and tools to cope with organisational change with respect to processes such as digitalisation. In addition, it is important to emphasise equal involvement in the workplace by applying an integrative approach to the healthcare workforce.

## Limitations and suggestions for further research

The online format for the interviews may be a limitation, as it may yield some disadvantages, such as being unable to observe the participant’s body language and emotional cues (Cater, 2011). However, face-to-face and online videoconferencing interviews have previously been found to yield the same interview quality (Cabaroglu *et al.*, 2010; Deakin and Wakefield, 2014), and the online method is also believed to be cost-effective (Cater, 2011).

It has been suggested that having two researchers conduct interviews is associated with more comprehensive data collection (Velardo and Elliott, 2021). In this study, however, only one researcher conducted the interviews. Moreover, one interviewee also participated in the quality café. Therefore, the insights shared during the quality café sessions may have influenced the information conveyed in this specific interview. Furthermore, the widely accepted guideline for determining an interview sample size is saturation, rooted in grounded theory (Glaser and Strauss, 2006). However, the sample size in this context emerged iteratively, aligning with the core principle of grounded theory as a dynamic process during research. Accordingly, we concluded that conducting 20 interviews was adequate to achieve data saturation, and we observed limited opportunities to form new categories (Hennink and Kaiser, 2022).

Regarding quantitative data collection, prior studies have pointed out that online questionnaires may yield fewer responses than paper questionnaires (Lefever *et al.*, 2007). However, online questionnaires may be preferable because they are time- and cost-effective (Ebert *et al.*, 2018). The response rate in this study was slightly higher than the general response rate (Wu *et al.*, 2022). Developing a questionnaire based on qualitative interview findings may generate high-quality items and improve content validity (McKenna *et al.*, 2004).

Factor analysis as an analytical method requires several considerations. For instance, researchers should consider sample size, factor extraction method and rotation method (Schmitt, 2011). These issues are addressed in this study.

Further research should focus on the future needs of medical secretaries with respect to workplace climate, perceived health parameters such as stress and overall workplace well-being, as well as how digitalisation as an organisational change may influence their professional role in healthcare. With reference to organisational change, it would be valuable to further explore the associations between digitalisation and health, and their mechanism.

## Figures and Tables

**Table 1:** Themes and categories from the qualitative data

Digitalisation as part of workplace change		A changing role of the medical secretary?	
Paving the way for digitalisation	Acknowledgment and self-empowerment	Digitalisation as an enabler	Thoughts and fears

**Source(s):** Authors work

**Table 2:** Descriptive statistics

Workplace		Full sample	Primary care and rehabilitation	Psychiatric care	Specialist care	Other
Number		N = 181	N = 52 (52 %)	N = 21 (12 %)	N = 107 (60 %)	N = 1 (1 %)
Age	35 years or younger	28	3	4	20	1
	36–50 years	55	12	7	36	0
	51 years or older	98	37	10	51	0

**Source(s):** Authors work

**Table 3:** Items, factor loadings (loading items), dimensions and total of variance explained

Factors (*number of loading items*)								
Item	1 (*6*)	2 (*5*)	3 (*6*)	4 (*5*)	5 (*4*)	6 (*3*)	7 (*3*)	Dimension
Forward planning and implementation workplace digitalisation	*0.948*	−0.057	0.091	0.054	0.063	0.023	−0.069	*Digital inclusiveness:* To be included in implementation of digitalisation initiatives
Assessment and evaluation of digitalisation initiatives	*0.912*	−0.041	0.017	0.006	0.117	−0.004	−0.083	
Provided information regarding digitalisation initiatives	*0.840*	0.104	−0.078	0.070	−0.072	−0.055	0.078	
Workplace digitalisation: participation and involvement	*0.712*	−0.032	0.066	0.050	0.062	0.136	−0.342	
Clear purposes of digitalisation implementation	*0.643*	0.099	0.001	−0.061	−0.085	−0.003	0.259	
Possible changes for the profession due to digitalisation	*0.465*	0.140	−0.192	0.040	0.131	0.192	−0.094	
Efficiency of education	−0.022	*0.953*	−0.080	0.089	−0.032	0.084	0.013	*Educational aspects:* To have knowledge and time set aside for education
Sufficient education	−0.057	*0.885*	−0.116	0.202	0.069	0.124	0.056	
Time allotted to education	0.024	*0.808*	0.256	−0.108	−0.028	−0.021	−0.121	
Adequate support and help	0.150	*0.632*	0.002	0.114	0.064	−0.028	0.100	
Sufficient time allotted to education	0.198	*0.612*	0.262	−0.210	−0.044	−0.276	−0.162	
Digitalisation may promote the development of medical secretaries	−0.190	0.128	*0.945*	−0.009	0.071	0.088	−0.130	*Positive impact of digitalisation:* Increased opportunities in a digitized workplace
Digitalisation as a career enabler	−0.241	0.240	*0.790*	0.048	0.015	0.126	−0.055	
Digitalisation as an opportunity	0.124	−0.082	*0.684*	0.274	−0.006	−0.005	0.080	
Greater variety among workplace tasks due to digitalisation	0.375	−0.201	*0.618*	−0.061	−0.123	0.038	−0.102	
Digitalisation as workplace facilitator	0.207	−0.010	*0.556*	−0.149	0.042	−0.070	0.301	
I am positive towards digitalisation	0.174	0.052	*0.457*	0.328	−0.087	−0.038	0.105	
Concerns and fears due to digitalisation	−0.046	−0.004	−0.114	*−0.819*	−0.080	0.055	−0.028	*Worries and concerns:* Thoughts and fears about digitalisation and the future role of medical secretaries
My profession is “threatened” by digitalisation	0.045	0.023	−0.374	*−0.697*	0.036	0.077	−0.125	
Thoughts about the future professional role	−0.081	−0.119	0.116	*−0.625*	0.042	0.027	0.042	
Digitalisation has changed my views on the profession	0.019	−0.012	0.230	*−0.458*	0.079	−0.026	0.100	
The future is bright	0.105	0.096	0.193	*0.427*	0.103	0.011	−0.030	
A promoting workplace climate regarding interprofessional collaboration	0.015	−0.159	0.062	0.042	*0.905*	0.011	0.200	*Workplace inclusion:* To feel included and acknowledged at the workplace
To be included and acknowledged	0.057	0.004	0.076	−0.085	*0.816*	0.059	−0.020	
Workplace hierarchy	0.014	−0.151	0.251	−0.125	*−0.589*	0.103	−0.071	
Equality and inclusiveness among professions	0.146	0.087	0.109	−0.146	*0.586*	−0.084	0.029	
Expectations to have knowledge regarding digitalisation	0.131	0.058	0.056	−0.062	−0.013	*0.835*	0.032	*Added responsibilities due to digitalisation:* New functions of medical secretaries
Expectations of forwarding knowledge regarding digitalisation	−0.001	0.041	0.190	−0.104	−0.039	*0.778*	−0.056	
Digitalisation tasks as a burden	−0.049	−0.236	−0.035	0.121	−0.043	*0.383*	0.271	
Stress due to new tasks	−0.170	−0.096	0.044	0.061	0.127	0.015	*0.577*	*Workday routines:* Perceived stress and workplace routines
Workplace changes due to digitalisation	−0.028	0.161	0.180	−0.260	0.004	0.132	*0.403*	
Unchanged everyday work	0.298	0.096	−0.285	−0.049	0.035	0.013	*0.394*	
*Eigenvalues*	11.612	3.223	2.689	2.079	1.546	1.316	1.003	
*Variance explained*	33.350	8.500	6.799	5.049	3.687	2.863	2.040	

**Source(s):** Authors work

**Table 4:** Spearman’s rank correlation coefficient analysis (Spearman’s rho)

Correlations (Spearman’s rho)					
	Factor 1	Factor 2	Factor 5		
Perceived health index	0.298**	0.325**	0.384**		

	Factor 1	Factor 2	Factor 3	Factor 5	Factor 7
Sense of coherence index	0.365**	0.406**	0.315**	0.365**	0.347**

**Note(s):** **Correlation is significant at a 0.001 level

**Source(s):** Authors work
